# Profiling gene alterations in striatonigral neurons associated with incubation of methamphetamine craving by cholera toxin subunit B-based fluorescence-activated cell sorting

**DOI:** 10.3389/fncel.2025.1542508

**Published:** 2025-02-12

**Authors:** Rachel D. Altshuler, Megan A. M. Burke, Kristine T. Garcia, Kenneth Class, Raffaello Cimbro, Xuan Li

**Affiliations:** ^1^Department of Psychology, University of Maryland College Park, College Park, MD, United States; ^2^Department of Cell Biology and Molecular Genetics, University of Maryland College Park, College Park, MD, United States; ^3^Division of Rheumatology, Johns Hopkins University School of Medicine, Baltimore, MD, United States; ^4^Program in Neuroscience and Cognitive Science, University of Maryland College Park, College Park, MD, United States

**Keywords:** methamphetamine craving, cholera toxin subunit B, fluorescence-activated cell sorting, striatonigral projection neurons, gene expression

## Abstract

**Introduction:**

In both rats and humans, methamphetamine (Meth) seeking progressively increases during abstinence, a behavioral phenomenon termed “incubation of Meth craving”. We previously demonstrated a critical role of dorsal striatum (DS) in this incubation in rats. However, circuit-specific molecular mechanisms in DS underlying this incubation are largely unknown. Here we combined a newly developed fluorescence-activated sorting (FACS) protocol with fluorescence-conjugated cholera toxin subunit B-647 (CTb-647, a retrograde tracer) to examine gene alterations in the direct-pathway (striatonigral) medium spiny neurons (MSNs) associated with incubation of Meth craving.

**Methods:**

We injected CTb-647 bilaterally into substantia nigra before or after training rats to self-administer Meth or saline (control condition) for 10 days (6 h/d). On abstinence day 1 or day 28, we collected the DS tissue from both groups for subsequent FACS and examined gene expressions in CTb-positive (striatonigral MSNs) and CTb-negative (primarily non-striatonigral MSNs). Finally, we examined gene expressions in DS homogenates, to demonstrate cell-type specificity of gene alterations observed on abstinence day 28.

**Results:**

On abstinence day 1, we found mRNA expression of *Gabrb3* decreased only in CTb-positive (but not CTb-negative) neurons of Meth rats compared with saline rats, while mRNA expression of *Usp7* decreased in all sorted DS neurons. On abstinence day 28, we found increased mRNA expression for *Grm3, Opcml*, and *Usp9x* in all sorted DS neurons, but not DS homogenate.

**Discussion:**

Together, these data demonstrated that incubation of Meth craving was associated with time-dependent, circuit-specific, and cell type-specific gene alterations in DS involved in glutamatergic, GABAergic, opioidergic, and protein degradation signaling.

## Introduction

Relapse is a key challenge for treating methamphetamine (Meth) addiction, for which no pharmacotherapy approved by Food and Drug Administration is available (Elkashef et al., 2008; Karila et al., 2010; Megan et al., 2023). In both rats and humans, cue-induced Meth seeking progressively increases after abstinence from Meth use (Shepard et al., 2004; Wang et al., 2013; Adhikary et al., 2017). Our prior work has demonstrated a critical role of dorsal striatum (DS) in this incubation of Meth craving in rats (Li et al., 2015). Specifically, we showed that cue-induced Meth seeking after prolonged abstinence is associated with DS activation, assessed by neuronal activity marker Fos. Furthermore, blockade of dopamine 1 receptor (D1R) signaling in DS decreased Meth seeking after prolonged, but not short abstinence (Li et al., 2015).

Previous work has also explored molecular mechanisms in DS associated with prolonged abstinence from extended-access Meth self-administration (Schwendt et al., 2012; Krasnova et al., 2013; Li et al., 2015; Daiwile et al., 2024), a behavioral procedure that leads to robust incubation of drug craving in rats (Lu et al., 2004). Most of these studies used the DS homogenate and showed alterations of mRNA or protein expressions of candidate targets (e.g., glutamate receptors or epigenetic enzymes) in DS homogenate after prolonged abstinence from Meth self-administration. In addition, using fluorescence-activated cell sorting (FACS), we found a selective increase of several candidate genes in Fos-positive DS neurons (activated by cue-induced Meth seeking after prolonged abstinence) compared with Fos-negative neurons (Li et al., 2015). While these studies uncovered the molecular mechanisms within DS associated with Meth relapse, circuit-specific molecular mechanisms underlying incubation of Meth craving, which could be critical for the action of DS to its downstream projection regions, have not been explored.

Over 95% of DS neurons are GABAergic projection medium spiny neurons (MSNs), which are divided into two major subpopulations based on their circuit specificities. The direct-pathway MSNs, which primarily express D1R, project to internal globus pallidus (Gpi) and substantia nigra pars reticulata (SNr). The indirect-pathway MSNs, which primarily express dopamine 2 receptor (D2R), project to external globus pallidus (Gpe) (Deng et al., 2006; Lobo, 2009). To examine circuit-specific molecular mechanisms in DS associated with incubation of Meth craving, here we combined a newly-developed FACS protocol with fluorescence-conjugated cholera toxin subunit B (CTb-647, a retrograde tracer, injected into SN), to measure the mRNA expression of candidate genes in striatonigral projections neurons (the direct-pathway MSNs) in DS after 1-day (low Meth seeking) or 28-day (incubated Meth seeking) abstinence from Meth self-administration. We focused on the direct-pathway MSNs because previous reports have demonstrated a critical role of D1R signaling in DS in Meth relapse across different animal models (Rubio et al., 2015; Li et al., 2015; Caprioli et al., 2017). It is of note that although previous studies demonstrated distinct roles of subregions of DS (dorsomedial and dorsolateral striatum) in drug-seeking behavior (Bossert et al., 2009; Wang et al., 2010; Corbit et al., 2012; Murray et al., 2012; Rubio et al., 2015; Caprioli et al., 2017), we found no sub-region specificity of DS in their roles in Meth seeking after prolonged abstinence (Li et al., 2015). Therefore, we focused on the entire DS in the current study.

We assessed whether there were time-dependent changes in mRNA expression of 33 candidate genes in striatonigral neurons during incubation of Meth craving. These genes were divided into four classes. The first class includes glutamate receptors because previous work in rodents implicated glutamate signaling across multiple brain areas in incubation of Meth craving (e.g., Li et al., 2015; Scheyer et al., 2016; Murray et al., 2019; Pena-Bravo et al., 2019). The second includes GABAergic receptors, based on previous work demonstrating the critical roles of GABAergic signaling in DS in context-induced reinstatement of Meth seeking (Rubio et al., 2015) and Meth-induced conditioned place preference (Jiao et al., 2016) in rats. Gene variations of GABAergic receptors have also been linked to Meth dependence in females (Xie et al., 2023). The third class includes opioid receptors and opioid-binding protein/cell adhesion molecules because previous work in rodents implicated opioidergic transmission in Meth-induced neuronal activation in DS and Meth-induced behavioral sensitization (Tien and Ho, 2011). The fourth class includes multiple ubiquitin-specific peptidases (USPs), which belong to the family of deubiquitinating enzymes and regulate synaptic function by controlling the specificity of protein degradation (Ciechanover, 2005; Patrick, 2006; Mabb and Ehlers, 2010; Bingol and Sheng, 2011). The roles of USPs in addiction-associated behavior are largely unknown, but a recent study demonstrated that systemic inhibition of ubiquitin-specific peptidase 7 (USP7) abolished cocaine-induced locomotor sensitization (Cheron et al., 2023). Finally, to examine whether differentially expressed genes (DEGs) are specific to isolated striatonigral MSNs (CTb-positive neurons) in DS, we measured mRNA expressions of these DEGs in both CTb-negative neurons (primarily non-striatonigral MSNs) and DS homogenate after abstinence from saline or Meth self-administration.

## Materials and methods

### Subjects

We used male (Charles River Laboratories; total *n* = 50) Sprague Dawley rats, including one rat for Experiment 1, 17 rats (Saline: *n* = 8, Meth: *n* = 9) for Experiment 2, 18 rats (Saline: *n* = 9, Meth: *n* = 9) for Experiment 3, and 10 rats (Saline: *n* = 5, Meth: *n* = 5) for Experiment 4. We excluded four rats due to health-related issues (*n* = 3) or failure to acquire stable Meth self-administration (*n* = 1). The male rats weighed 300–400 g before surgery. Rats were maintained under a reverse 12 h light/dark cycle with food and water available *ad libitum*, housed four per cage before surgery, and then single-housed after surgery. We performed the experiments under the protocols approved by the University of Maryland College Park Animal Care and Use Committee and in accordance with the Guide for the Care and Use of Laboratory Animals (National Institute of Health).

### Intravenous surgery

Rats were anesthetized with isoflurane gas (5% induction; 2–3% maintenance), and silastic catheters were inserted into each rat’s jugular vein as previously described (Li et al., 2013; Li et al., 2018). We injected the rats with ketoprofen (2.5 mg/kg) after surgery to relieve pain and inflammation and allowed the rats to recover for 5–7 days before the start of self-administration training. During the recovery and training phases, catheters were flushed every 24–48 h with gentamicin (5 mg/mL) dissolved in saline.

### Apparatus

We trained the rats in self-administration chambers located inside sound-attenuating cabinets and controlled by a Med Associates system (Georgia, VT). Each chamber has two levers located 8–9 cm above the floor. During self-administration training, presses on the retractable (active) lever activated the infusion pump, which delivered saline/Meth infusions; presses on the stationary (inactive) lever were not reinforced. For intravenous infusions, we connected each rat’s catheter to a liquid swivel *via* polyethylene-50 tubing protected by a metal spring. We then attached the liquid swivel (Instech) to a 20-mL syringe *via* polyethylene-50 tubing and to a 22-gauge modified needle (Plastics One, VA).

### Meth (saline) self-administration training

We used an extended-access training procedure as described previously (Li et al., 2018; Li et al., 2019). We trained the rats to self-administer saline or Meth (kindly provided by National Institute on Drug Abuse Drug Supply Program) under a fixed-ratio 1 (FR1) with a 20-s time-out reinforcement schedule. Each training session included six 1-h sessions with 10 min off in between sessions. We dissolved Meth in saline, and the rats self-administered Meth at a dose of 0.1 mg/kg/infusion over 3.5 s (0.10 mL/infusion). We trained the rats for 10 sessions over an 11-day period (off day on the fifth or sixth day). We used Brevital (3–4 mg/kg) to check the functionality of the catheter for low responders during the training period.

The sessions started at the onset of the dark cycle and began with the extension of the active lever and the illumination of the red house light, which remained on for the duration of the session. During training, active lever presses led to the delivery of a Meth/saline infusion and a compound 5-s tone/light cue [the tone and light modules (Med Associates) were located above the active lever]. During the 20-s time-out, we recorded non-reinforced lever presses. To prevent overdose, we set 90 infusions as the maximum for the 6-h training session, with 15 infusions as the maximum for each 1-h session. After the rats received the maximum infusions or at the end of the 6-h training session, the red house light was turned off and the active lever was retracted. Rats were taken out of the operant chamber and returned to their home cages daily after each 6-h training session.

### Abstinence phase

During this phase, rats were individually housed in the animal facility and handled two to three times per week.

### Cholera toxin subunit B-Alexa Fluor 647 conjugate (CTb-647) injections into SN

**FIGURE 1:**
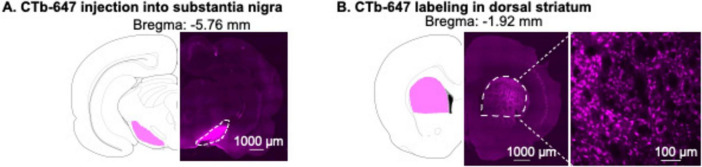
Validation of CTb-647 injection site in substantia nigra (SN) and CTb-647 labeling in dorsal striatum (DS). **(A)** A representative image of CTb-647 injection site in SN. **(B)** A representative image of CTb-647 labeling in DS of the same rat.

We injected CTb-Alexa Fluor 647 conjugate (CTb-647, Cat# C34778, ThermoFisher Scientific) dissolved in phosphate-buffered saline (4 μg/μL) (Mandelbaum et al., 2019), bilaterally into SN (400 nl/side). CTb-647 was delivered by a 10-μL, 33-gauge Nanofil syringe attached to UltraMicroPump (UMP3) with Sys-Micro4 Controller (World Precision Instruments) at a rate of 80 nl/min. We then left the needle in place for an additional 5 min to allow diffusion. The coordinates for SN were as follows; anterior-posterior (AP): −5.3 mm, medial-lateral (ML): ± 2.5 mm, dorsal-ventral (DV): −8.5 mm (Deng et al., 2006). Representative images of CTb-647 injection site in the SN and CTb-647 retrograde labeling in DS are shown in Figures 1A,B.

### CTb-647 histology

Rats were anesthetized with isoflurane and perfused transcardially with ∼100 mL of 0.1 M phosphate-buffered saline (PBS), followed by 400 mL of 4% paraformaldehyde (PFA) in PBS. We extracted the brain, which was then postfixed in 4% PFA for 2 h before being transferred to 30% sucrose in PBS for 48 h at 4°C. After freezing the brains on dry ice, we cut serial coronal sections (40 μm) using a Leica Microsystems cryostat. After rinsing sections in PBS, we mounted the sections onto glass slides (Basix™ Adhesion Microscope Slides, Cat #23-888-115). After air drying, sections were coverslipped with Fluormount G (Electron Microscopy Sciences).

### Image acquisition

For Experiment 1, we digitally captured dark-field images of CTb-647 in SN and DS using a Hamamatsu Flash 4.0 LT Plus camera attached to the Nikon Ti2 microscope.

### FACS

We dissected DS from a 2-mm coronal section (between approximately Bregma AP +2.28 and +0.36 mm) (Paxinos and Watson, 2005) using a brain matrix (ASI instruments). We froze the brain tissue in microcentrifuge tubes on dry ice and stored the tissue at −80°C. To sort CTb-positive and CTb-negative neurons from frozen tissue, we used a modified protocol based on previous studies (Rubio et al., 2016; Martin et al., 2017). Briefly, we placed the microcentrifuge tube containing the frozen tissue on ice and fixed the tissue with 1 mL 2% PFA (diluted from a 16% PFA stock solution, Cat # 15170-S, Electron Microscopy Science) for 40 min on ice. Next, we quickly washed the tissue twice with 1 mL PBS. To remove any residual PFA, we incubated the tissue with PBS at 4°C for 10 min with end-over-end mixing and rinsed the tissue again with PBS.

We transferred the tissue from the microcentrifuge tube onto the cold glass plate on ice. We minced the tissue with razor blades 15 times for each orthogonal direction and then transferred the tissue into 0.6 mL cold PBS. We triturated the tissue with an 18-gauge needle 5–8 times, then centrifuged the tissue at 370 g for 2 min (4°C). After discarding the supernatant, we added 1 mL cold Accutase (Cat # SCR005, Millipore) to the pellet and mixed it by pipetting up and down 4 times. We then incubated the tissue for 30 min at room temperature with end-over-end mixing. The tissue was centrifuged at 830 g for 2 min (4°C). After discarding the supernatant, we re-suspended the pellet in 0.6 mL cold PBS. We triturated each tissue sample twice in series using 23-gauge and 25-gauge needles. Each trituration step consisted of triturating up and down fifteen times, followed by 5 min on ice to sediment the larger debris and un-dissociated cells. We combined the supernatant from each trituration step in a total volume of 1.2 mL. We filtered the supernatant with a 100-μm strainer, followed by a 40-μm cell strainer (Falcon brand, BD Biosciences). After collecting the cells by centrifugation (1,125 g, 3 min, 4°C), we re-suspended the cells with 0.5 mL cold PBS and proceeded to cell staining.

We incubated the cells with PE-labeled anti-NeuN antibody (1:500, FCMAB317PE, Millipore, RRID:AB_10807694) for 30 min at 4°C and added DAPI (1 μg/mL, Cat # FCMAB317PE, Millipore Sigma) during the last 5-min of the incubation period. We then washed the cells by adding 0.8 mL cold PBS and centrifuged the cells (1,125 g, 3 min, 4°C). The cells were washed again with 1 mL cold PBS, followed by centrifugation (1,125 g, 3 min, 4°C), and resuspended with 0.5 mL cold PBS. We filtered the cells again through a 40-μm filter before sorting in a FACSAria II cell sorter (BD Sciences).

**FIGURE 2:**
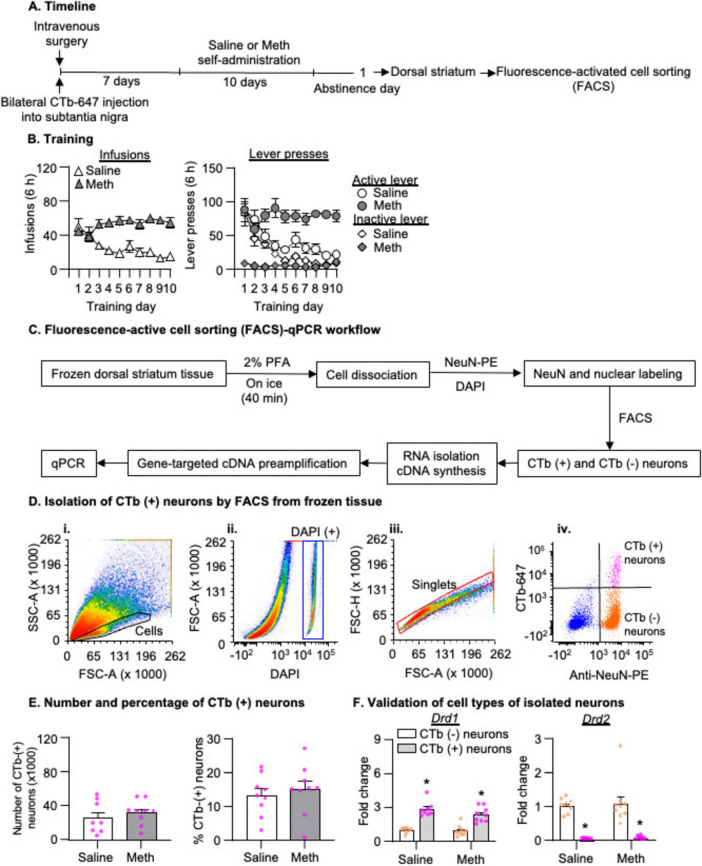
Isolation of striatonigral projection neurons by fluorescence-activated cell sorting (FACS) after 1-day abstinence from saline or methamphetamine (Meth) self-administration. **(A)** Experimental timeline for Experiment 2. **(B)** Saline and Meth self-administration. Data are mean ± SEM number of saline of Meth (0.1 mg/kg/infusion) infusions or lever presses during the ten 6-h daily self-administration sessions. Saline: *n* = 8; Meth: *n* = 9. **(C)** FACS-qPCR workflow. **(D)** Gating strategy for FACS of CTb-positive neurons (striatonigral projection neurons) by FACS. (i,ii) Density plots show the cell gate based on the area of forward scatter (FSC-A), the area of side scatter (SSC-A) properties, and DAPI-positive signals. (iii) A density plot shows the singlet gate based on the area and height of FCS within the DAPI-positive gate. (iv) A scatter plot of events within the singlet gate shows FACS of NeuN-positive (x-axis) and CTb-positive or CTb-negative (y-axis) cells (striatonigral or non-striatonigral neurons) in dorsal striatum. CTb-positive and CTb-negative neurons are in the top right and bottom right quadrants, respectively. **(E)** Number and percentage of CTb-positive neurons (out of the total number of neurons) isolated by FACS in saline and Meth rats. Saline: *n* = 8; Meth: *n* = 9. **(F)** *Drd1* and *Drd2* mRNA expression in CTb-positive and CTb-negative neurons. Data are presented as folds of mean values in CTb-negative neurons, and error bars indicate SEM. *Different from CTb-negative neurons, *p* < 0.001, Saline: *n* = 8; Meth: *n* = 9.

As previously reported (Li et al., 2015; Rubio et al., 2016; Li et al., 2019), neurons can be identified based on the distinct forward (FSC) and side (SSC) scatter properties (Figure 2D). After defining the cell population by the presence of DAPI positive events, we gated single cells by the area and height of FCS and conducted subsequent sorting within this single-cell population. We sorted neurons according to PE (NeuN-immunopositive) and Alexa Fluor 647 (CTb-647) fluorescence signals. We set the threshold of Alexa Fluor 647 fluorescence signal based on background fluorescence signals of NeuN-negative populations. We collected NeuN-positive+CTb-positive events (CTb-positive neurons) and NeuN-postive+CTb-negative events (CTb-negative neurons). The FACS data were analyzed by using FCS Express 6 (De Novo Software, Glendale, CA).

### RNA extraction, cDNA synthesis, and qPCR from FACS-isolated neurons

We collected sorted cells directly into 50 μL of the extraction buffer from the PicoPure RNA isolation kit (Cat # KIT0204, Applied Biosystems) and lysed the cells by pipetting up and down 10 times, followed by incubating for 30 min at 42°C. After centrifuging the suspension at 2,300 g at 4°C for 2 min, we collected the supernatant for RNA extraction. We extracted the RNA using PicoPure RNA isolation kit and synthesized single-strand cDNA using the Superscript III First-Strand Synthesis Kit (Cat # 18-080-051, Fisher Scientific) according to the manufacturers’ protocol.

We used gene-targeted preamplification of cDNA as described previously (Li et al., 2015; Li et al., 2019). Briefly, we used pooled primer solution with 0.2X concentration of TaqMan ABI primer/probes (20X TaqMan gene expression assay as the stocking solution) and 80 nM of customized primer sets (Table 1). Each cDNA sample (7.5 μL) was mixed with 7.5 μL of the pooled primer solution and 15 μL of TaqMan PreAmp Master Mix (Cat # 4391128, Thermo Fisher). We preamplified cDNA in a MJ Mini Thermal Cycler (Cat # PTC1148EDU, Bio-Rad) using the following program: 95°C hold for 10 min, denaturation at 90°C for 15 s, and annealing and extension at 60°C for 4 min (14 cycles). We diluted the preamplified cDNA product seven times before performing quantitative PCR (qPCR). We performed qPCR in duplicates with a Fam-labeled probe for each target gene and a Vic-labeled probe for the endogenous control gene (*NeuN*). We used TaqMan Advanced Fast PCR Master Mix (Cat # 4444965, Thermo Fisher) in a Bio-Rad CFX96 system using the following program: 95°C hold for 20 s, then 40 cycles with denaturation at 95°C for 3 s, and annealing and extension at 60°C for 30 s. We analyzed reactions using the ΔΔCt method with *NeuN* as the housekeeping gene.

**TABLE 1:** Primer/probe sequences.

Full names	Known as	TaqMan probe or primer/probe	Forward primer	Reverse primer
**Dopaminergic signaling**				
*Dopamine receptor D1*	*Drd1*	Rn03062203_s1	NA	NA
*Dopamine receptor D2*	*Drd2*	Rn00561126_m1	NA	NA
**GABAergic signaling**				
*Gamma-aminobutyric acid type B receptor subunit 2*	*Gabbr2*	Rn00582550_m1	NA	NA
*Gamma-aminobutyric acid type A receptor subunit alpha1*	*Gabra1*	Rn00788315_m1	NA	NA
*Gamma-aminobutyric acid type A receptor subunit alpha3*	*Gabra3*	Rn00567055_m1	NA	NA
*Gamma-aminobutyric acid type A receptor subunit alpha5*	*Gabra5*	Rn00568803_m1	NA	NA
*Gamma-aminobutyric acid type A receptor subunit beta2*	*Gabrb2*	Rn00564149_m1	NA	NA
*Gamma-aminobutyric acid type A receptor subunit beta3*	*Gabrb3*	Rn00567029_m1	NA	NA
*Gamma-aminobutyric acid type A receptor subunit gamma2*	*Gabrg2*	Rn01464079_m1	NA	NA
**Housekeeping genes**				
*Glyceraldehyde-3-phosphate dehydrogenase*	*Gapdh*	CTCATGACCACAGTCCA	GACAACTTTGGCATC GTGGAA	CACAGTCTTCTGAGTGG CAGTGA
*RNA binding fox-1 homolog 3*	*NeuN*	CACTCCAACAGCGTGAC	GGCCCCTGGCAGA AAGTAG	TTCCCCCTGGTCC TTCTGA
**Glutamatergic signaling**				
*Glutamate ionotropic receptor AMPA type subunit 1*	*Gria1*	Rn00709588_m1	NA	NA
*Glutamate ionotropic receptor AMPA type subunit 2*	*Gria2*	Rn00568514_m1	NA	NA
*Glutamate ionotropic receptor AMPA type subunit 3*	*Gria3*	Rn00583547_m1	NA	NA
*Glutamate ionotropic receptor AMPA type subunit 4*	*Gria4*	Rn00568544_m1	NA	NA
*Glutamate ionotropic receptor NMDA type subunit 1*	*Grin1*	Rn01436038_m1	NA	NA
*Glutamate ionotropic receptor NMDA type subunit 2a*	*Grin2a*	Rn00561341_m1	NA	NA
*Glutamate ionotropic receptor NMDA type subunit 2b*	*Grin2b*	Rn00680474_m1	NA	NA
*Glutamate metabotropic receptor 1*	*Grm1*	Rn01440619_m1	NA	NA
*Glutamate metabotropic receptor 2*	*Grm2*	Rn01447672_m1	NA	NA
*Glutamate metabotropic receptor 3*	*Grm3*	Rn01755349_m1	NA	NA
*Glutamate metabotropic receptor 4*	*Grm4*	Rn01428450_m1	NA	NA
*Glutamate metabotropic receptor 5*	*Grm5*	Rn00690337_m1	NA	NA
**Opioidergic signaling**				
*Opioid-binding protein/cell adhesion molecule*	*Opcml*	Rn00587759_m1	NA	NA
*Opioid receptor delta 1*	*Oprd1*	Rn07310941_m1	NA	NA
*Opioid receptor kappa 1*	*Oprk1*	Rn00567737_m1	NA	NA
*Opioid receptor mu 1*	*Oprm1*	Rn00565144_m1	NA	NA
**Ubiquitin specific peptidase (protein degradation signaling)**				
*Ubiquitin specific peptidase 5*	*Usp5*	Rn01492298_m1	NA	NA
*Ubiquitin specific peptidase 7*	*Usp7*	Rn01169349_m1	NA	NA
*Ubiquitin specific peptidase 9x*	*Usp9x*	Rn01430666_m1	NA	NA
*Ubiquitin specific peptidase 11*	*Usp11*	Rn01430793_m1	NA	NA
*Ubiquitin specific peptidase 14*	*Usp14*	Rn01236255_m1	NA	NA
*Ubiquitin specific peptidase 15*	*Usp15*	Rn00595467_m1	NA	NA
*Ubiquitin specific peptidase 22*	*Usp22*	Rn01773117_m1	NA	NA
*Ubiquitin specific peptidase 34*	*Usp34*	Rn01231137_m1	NA	NA
*Ubiquitin specific peptidase 47*	*Usp47*	Rn00623739_m1	NA	NA
*Ubiquitin specific peptidase 48*	*Usp48*	Rn01640090_m1	NA	NA

We chose *NeuN* as the housekeeping gene for consistency with our previous studies (Li et al., 2015; Li et al., 2019). We also verified the uniformity of the pre-amplification step by comparing cDNA templates from the unamplified and pre-amplified samples. All ΔΔCt values of the tested genes between pre-amplified and unamplified cDNA samples were within the range of ± 1.5 (data not shown).

### RNA extraction, cDNA synthesis, and qPCR from DS homogenates

We dissected and stored the DS tissue using the same procedure as described above. We extracted RNA from DS homogenates using the miRNeasy Mini Kit (Cat # 217004, Qiagen) and synthesized single-strand cDNA using the Superscript III First-Strand Synthesis Kit (Fisher Scientific, Cat # 18-080-051) according to the manufacturer’s protocol. We measured mRNA concentrations using Nanodrop and diluted cDNA 20 times before performing qPCR. We performed qPCR in duplicates with a Fam-labeled probe for each target gene and a Vic-labeled probe for the housekeeping gene, glyceraldehyde 3-phosphate dehydrogenase (*Gapdh*). We used TaqMan Advanced Fast PCR Master Mix (Thermo Fisher) in Bio-Rad CFX96 system using the following program: 95°C hold for 20 s, then 40 cycles with denaturation at 95°C for 3 s, and annealing and extension at 60°C for 30 s. We analyzed the reaction using the ΔΔCt method with *Gapdh* as the housekeeping gene.

### Experiment 1: validation of CTb-647 injection site in SN and retrograde labeling in DS

We injected CTb-647 ipsilaterally into SN of one male rat. One week later, we perfused the rat to validate CTb-647 as described above.

### Experiment 2: mRNA expression of candidate genes in striatonigral projection neurons after 1-day abstinence from saline or Meth self-administration

The experimental timeline of this experiment is illustrated in Figure 2A. We performed intravenous surgery on two groups of male rats and injected CTb into the SN immediately following the intravenous surgery. We trained the rats to self-administer Meth (*n* = 9) or saline (*n* = 8) as described above. On abstinence day 1, we performed live decapitations, collected DS, and froze the tissue. We then processed the tissue for FACS as described above, and collected CTb-positive + NeuN-positive cells (CTb-positive neurons) and CTb-negative + NeuN-positive cells (CTb-negative neurons). Next, we extracted the RNA, and performed cDNA synthesis, gene-targeted preamplification and qPCR. We compared mRNA expressions of several candidate genes between saline and Meth rats in CTb-positive and CTb-negative neurons.

### Experiment 3: mRNA expression of candidate genes in striatonigral projection neurons after 28-day abstinence from saline or Meth self-administration

The experimental timeline of this experiment is illustrated in Figure 4A. We performed intravenous surgery on two groups of male rats and trained them to self-administer either saline (*n* = 9) or Meth (*n* = 9) as described above. On abstinence day 18, we injected CTb-647 bilaterally into the SN. On abstinence day 28, we performed live decapitations, collected DS, and froze the tissue. We then processed the tissue for FACS as described above and collected CTb-positive + NeuN-positive cells (CTb-positive neurons) and CTb-negative + NeuN-positive cells (CTb-negative neurons). Next, we extracted the RNA, and performed cDNA synthesis, gene-targeted preamplification and qPCR. We compared mRNA expressions of several candidate genes between saline and Meth rats in CTb-positive and CTb-negative neurons, respectively.

### Experiment 4: mRNA expression of candidate genes in the DS homogenate after 28-day abstinence from saline or Meth self-administration

The experimental timeline of this experiment is illustrated in Figure 6A. To examine whether gene alternations observed in Experiment 3 were specific in sorted striatal neurons, we measured mRNA expression of these genes in DS homogenates on abstinence day 28. We performed intravenous surgery on two groups of male rats and trained them to self-administer either saline (*n* = 5) or Meth (*n* = 5) as described above. On abstinence day 28, we performed live decapitations, collected DS, and froze the tissue. We performed RNA extraction and cDNA synthesis, then used qPCR to quantify genes that were significantly altered in Experiment 3.

### Statistical analysis

We analyzed the behavioral and molecular data with SPSS (version 27), GraphPad (version 10), FCS Express (Version 6) using mixed ANOVAs or t-test, as appropriate. We used univariate ANOVAs to perform post hoc tests following significant main effects. For gene expression data, we excluded one outlier value above and/or below the threshold [defined by above or below three median absolute deviations (MADs) (Leys et al., 2013)] and samples with undetectable Ct values from the data presentation and statistical analysis. To correct for multiple *t*-tests comparing the mRNA expression of 33 genes in CTb-positive neurons between saline and Meth groups, we used the false discovery rate approach in Graphpad (Two-stage linear step-up procedure of Benjamini, Krieger and Yekutieli) and set the Q value at 15% (false discovery rate). Between- and within-subject factors of the analyses are indicated in the Results section, and all statistical comparisons are listed in Supplementary Table S1.

## Results

### Experiment 1: validation of CTb-647 injection site in SN and retrograde labeling in DS

A representative image shows the injection site of CTb-647 in SN (Figure 1A). One week after CTb-647 injections, we observed dense CTb-647 labeling in the dorsal striatum (Figure 1B).

### Saline or Meth self-administration (Experiments 2–4)

Rats in all experiments demonstrated escalation of Meth, but not saline, self-administration and showed a strong preference for the Meth-associated active lever over the inactive lever during the training phase (Figures 2B, 4B, 6B). Detailed statistical information is provided in Supplementary Table S1.

### Experiment 2: mRNA expression of candidate genes in striatonigral projection neurons in DS after 1-day abstinence from saline or Meth self-administration

#### FACS of striatonigral projection neurons in DS

We measured mRNA expression of candidate genes in FACS-isolated striatonigral neurons (CTb-647 labeled cells in DS as shown in Figure 1B) after 1-day abstinence, when Meth craving is low, from Meth or saline self-administration. In Figure 2C, we illustrated the pipeline of the FACS protocol. To ensure the retention of cytoplasmic markers like CTb-647 for subsequent FACS, we incubated the frozen striatal tissue with 2% PFA while the frozen tissue gradually thawed on ice for 40 min (see more in Discussion). In Figure 2D, we used one saline rat from Experiment 2 to illustrate representative FACS analysis. All events during FACS are shown in the light scattergrams as a density plot in which the forward-scatter (FSC, x-axis) indicates the size of particles and the side-scatter (SSC, y-axis) indicates the granularity of particles (Figure 2D,i). DNA-bearing cell populations were identified by DAPI staining (Figure 2D,ii). Next, we defined the “Singlets” gate from the “DAPI-positive” gate based on the height and area of FSC (Figure 2D,iii), to exclude cells that were not dissociated completely (e.g., doublets). Within the “Singlets” gate, we gated CTb-positive and CTb-negative neurons based on both immunolabeling of NeuN with anti-NeuN-PE antibody and CTb-647 signals. In this scatter plot (Figure 2D,iv), the purple and organge dots represent CTb-positive and CTb-negative neurons, respectively, and the blue dots represent all non-neuronal cells.

Applying the FACS analysis above to all rats in Experiment 2, we observed a similar number of CTb-positive neurons and a similar percentage of CTb-positive neurons out of the total number of neurons in the DS between saline and Meth groups (Figure 2E, *p* > 0.05). In addition, we found a significant enrichment of *Drd1* mRNA and a significant depletion of *Drd2* mRNA in CTb-positive neurons compared with CTb-negative neurons. This analysis, which includes the between-subjects factor of Drug (Saline, Meth) and the within-subject factor of Cell-type (CTb-negative, CTb-positive), showed a significant main effect of Cell type (Figure 2F, *Drd1*: *F*_1,15_ = 178.872, *p* < 0.001; *Drd2*: *F*_1,15_ = 67.335, *p* < 0.001) but no main effect of Drug or significant interaction between Drug and Cell type (*p* > 0.05). Together, these data validated that our FACS protocol successfully isolated the striatonigral projection neurons, which are primarily D1R-expressing neurons (Deng et al., 2006).

#### mRNA expression of candidate genes in striatonigral projection neurons after 1-day abstinence

**FIGURE 3:**
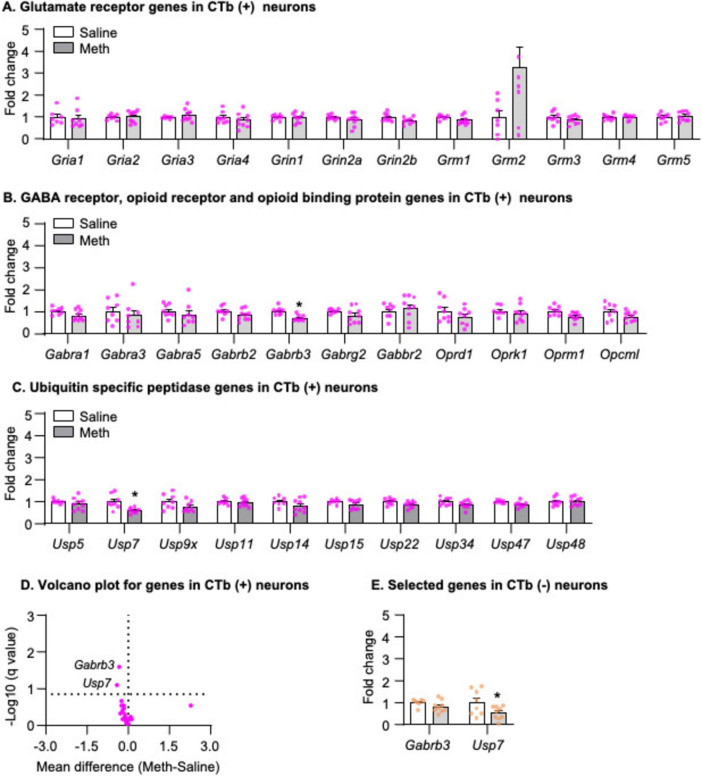
Gene expression in dorsal striatal neurons was isolated by fluorescence-activated cell sorting (FACS) after 1-day abstinence from saline or methamphetamine (Meth) self-administration. **(A–C)** Gene expression of glutamate receptors, GABA receptors, opioid receptors, opioid binding protein, and ubiquitin-specific peptidases in CTb-positive neurons. Data are presented as folds of mean values in CTb-positive neurons from saline rats, and error bars indicate SEM. *Different from CTb-positive neurons from saline rats, *p* < 0.05, Saline: *n* = 6–8; Meth: *n* = 8–9. **(D)** A volcano plot of mRNA expressions of all genes in CTb-positive neurons with the x-axis representing mean differences between Meth and saline rats (where the dashed vertical line is set as 0) and the y-axis representing -Log_10_ (q value) (where *q* ≤ 0.15 is considered significant; dashed horizontal line). **(E)** Gene expression of *Gabrb3 and Usp7* in CTb-negative neurons. Data are presented as folds of mean values in CTb-negative neurons from saline rats, and error bars indicate SEM. *Different from CTb-negative neurons of saline rats, *p* < 0.05, Saline: *n* = 7–8; Meth: *n* = 9.

We analyzed the mRNA data using the between-subjects factor of Drug (Saline, Meth) and found that no glutamate receptor genes exhibited statistically significant changes (Figure 3A), but that two genes exhibited significantly decreased mRNA expression in striatonigral projection neurons of Meth rats compared with those of saline rats, including *Gabrb3* (Figure 3B, *t*_14_ = 4.275, *p* < 0.001) and *Usp7* (Figure 3C, *t*_14_ = 3.335, *p* = 0.005). The volcano plot in Figure 3D illustrated -Log_10_(q value) and mean differences between Meth and Saline for all candidate genes. Finally, to examine whether the decreases in mRNA expression of *Gabrb3* and *Usp7* are specific to striatonigral projection neurons, we analyzed the mRNA expression of these two genes in CTb-negative neurons and found that *Gabrb3* (Figure 3E, *p* > 0.05) exhibited no changes, while *Usp7* exhibited significantly decreased mRNA expression in Meth rats compared with saline rats (Figure 3E, *Usp7*: *t*_15_ = 2.200, *p* = 0.044).

In summary, using our newly developed retrograde-tracer-based FACS protocol, we successfully isolated striatonigral MSNs (CTb-positive neurons), in which we detected a significant enrichment and depletion of *Drd1* and *Drd2* mRNA expressions, respectively, compared with CTb-negative neurons. Furthermore, our results demonstrated that mRNA expression of *Gabrb3* exhibited a selective decrease in striatonigral neurons, while mRNA expression of Usp7 decreased in all neurons in Meth rats compared with saline rats on abstinence day 1.

To examine whether there were time-dependent changes in mRNA expression of candidate genes after prolonged abstinence, we measured the mRNA expression of these candidate genes in striatonigral projection neurons after 28-days abstinence, when Meth seeking is incubated, from Meth or saline self-administration.

### Experiment 3: mRNA expression of candidate genes in striatonigral projection neurons after 28-day abstinence from saline or Meth self-administration

#### FACS of striatonigral projection neurons in DS

**FIGURE 4:**
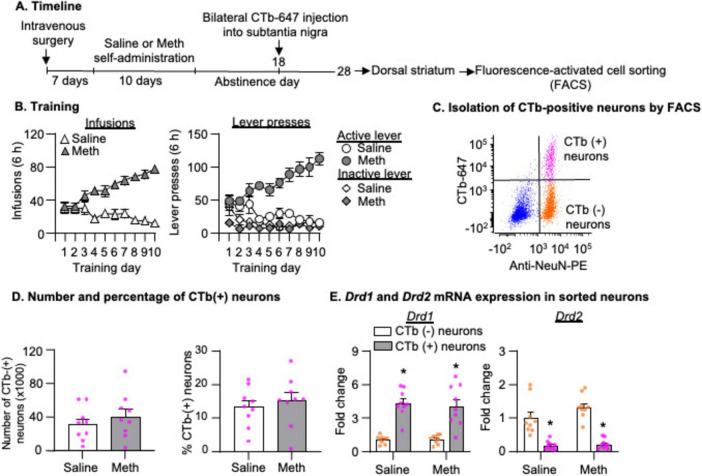
Isolation of striatonigral projection neurons by fluorescence-activated cell sorting (FACS) after 28-day abstinence from saline or methamphetamine (Meth) self-administration. **(A)** Experimental timeline for Experiment 2. **(B)** Saline and Meth self-administration. Data are mean ± SEM number of saline of Meth (0.1 mg/kg/infusion) infusions or lever presses during the ten 6-h daily self-administration sessions. Saline: *n* = 9; Meth: *n* = 9. **(C)** A scatter plot of events within the singlet gate shows FACS of NeuN-positive (x-axis) and CTb-positive or CTb-negative (y-axis) cells (striatonigral or non-striatonigral neurons) in dorsal striatum. CTb-positive and CTb-negative neurons are in the top right and bottom right quadrants, respectively. **(D)** Number and percentage of CTb-positive neurons (out of the total number of neurons) isolated by FACS in saline and Meth rats. Saline: *n* = 9; Meth: *n* = 9. **(E)** *Drd1* and *Drd2* mRNA expression in CTb-positive and CTb-negative neurons. Data are presented as folds of mean values in CTb-negative neurons, and error bars indicate SEM. *Different from CTb-negative neurons, *p* < 0.001, Saline: *n* = 9; Meth: *n* = 9.

Figure 4C shows a representative FACS analysis from a saline rat. Using the same analysis as described in Experiment 2, we observed a similar number of CTb-positive neurons and a similar percentage of CTb-positive neurons out of the total number of neurons in the DS between saline and Meth groups (Figure 4D, *p* > 0.05). The analysis of *Drd1* and *Drd2* mRNA showed a significant main effect of Cell type (Figure 4E, *Drd1*: *F*_1,16_ = 108.206, *p* < 0.001; *Drd2*: *F*_1,16_ = 99.526, *p* < 0.001) but no main effect of Drug or significant interaction between Drug and Cell type (*p* > 0.05). These data indicated a significant enrichment and depletion of *Drd1* and *Drd2* mRNA, respectively, in CTb-positive neurons, compared with CTb-negative neurons.

#### mRNA expression of candidate genes in striatonigral projection neurons after 28-day abstinence

**FIGURE 5:**
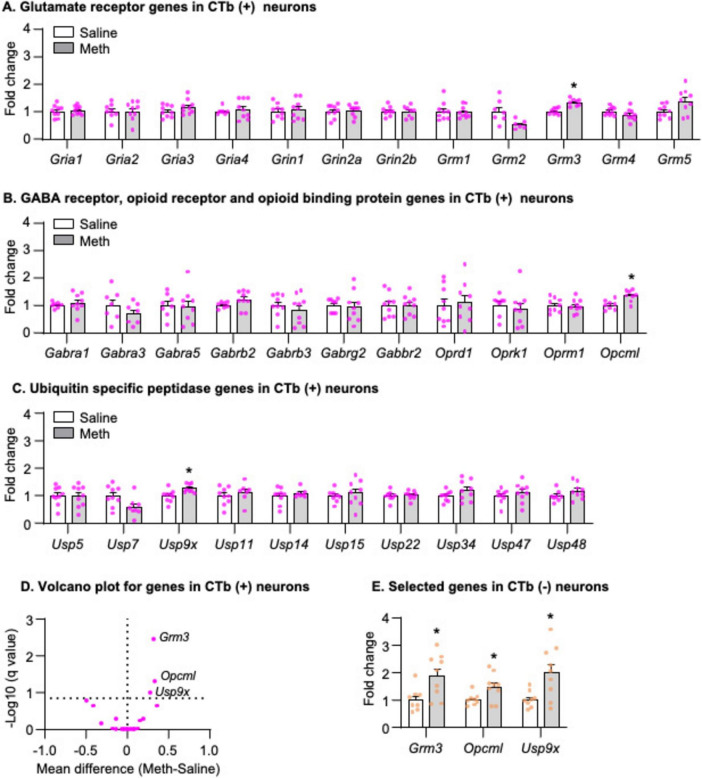
Gene expression in dorsal striatal neurons was isolated by fluorescence-activated cell sorting (FACS) after 28-day abstinence from saline or methamphetamine (Meth) self-administration. **(A–C)** Gene expression of glutamate receptors, GABA receptors, opioid receptors, opioid binding protein, and ubiquitin-specific peptidases in CTb-positive neurons. Data are presented as folds of mean values in CTb-positive neurons from saline rats, and error bars indicate SEM. *Different from CTb-positive neurons from saline rats, *p* < 0.05, Saline: *n* = 7–9; Meth: *n* = 8–9. **(D)** A volcano plot of mRNA expressions of all genes in CTb-positive neurons with the x-axis representing mean differences between Meth and saline rats (where the dashed vertical line is set as 0) and the y-axis representing -Log_10_ (*q* value) (where *q* ≤ 0.15 is considered significant; dashed horizontal line). **(E)** Gene expression of *Grm3*, *Opcml*, and *Usp9x* in CTb-negative neurons. Data are presented as folds of mean values in CTb-negative neurons from saline rats, and error bars indicate SEM. *Different from CTb-negative neurons from saline rats, *p* < 0.05, Saline: *n* = 8; Meth: *n* = 9.

We analyzed the mRNA data using the between-subjects factor of Drug (Saline, Meth) and found that three genes exhibited significantly increased mRNA expression in striatonigral projection neurons of Meth rats compared with those of saline rats, including *Grm3* (Figure 5A, *t*_14_ = 5.358, *p* < 0.001), *Opcm1* (Figure 5B, *t*_14_ = 3.602, *p* = 0.003), and *Usp9x* (Figure 5C, *t*_15_ = 2.995, *p* = 0.009). The volcano plot in Figure 5D illustrated -Log_10_(q value) and mean differences between Meth and Saline for all candidate genes. To examine whether the increases of *Grm3*, *Opcm1* and *Usp9x* mRNA expression were specific to striatonigral MSNs, we analyzed the mRNA expression of these three genes in CTb-negative neurons and found that all three genes also exhibited significantly increased mRNA expression in Meth rats compared with saline rats (Figure 5E, *Grm3*: *t*_15_ = 2.827, *p* = 0.013, *Opcm1*: *t*_15_ = 2.453, *p* = 0.027; *Usp9x*: t_15_ = 2.832, *p* = 0.013).

In summary, we found that mRNA expressions of *Grm3*, *Opcm1, and Usp9x* increased in both CTb-positive and CTb-negative neurons in Meth rats compared with saline rats on abstinence day 28.

### Experiment 4: mRNA expression of candidate genes in the DS homogenate after 28-day abstinence from saline or Meth self-administration

**FIGURE 6:**
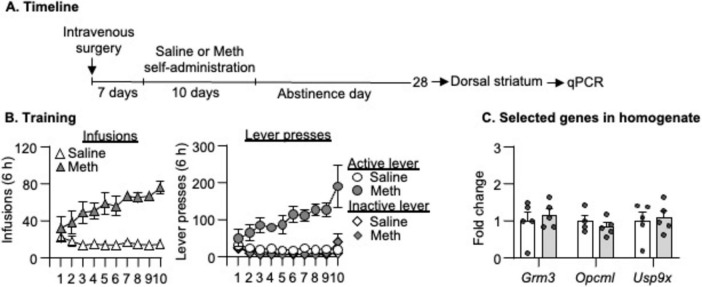
Gene expression in dorsal striatal homogenate after 28-day abstinence from saline or methamphetamine (Meth) self-administration. **(A)** Experimental timeline for Experiment 3. **(B)** Saline and Meth self-administration. Data are mean ± SEM number of saline of Meth (0.1 mg/kg/infusion) infusions or lever presses during the ten 6-h daily self-administration sessions. Saline: *n* = 5; Meth: *n* = 5. **(C)** Gene expression of *Grm3*, *Opcml*, and *Usp9x* in dorsal striatal homogenate. Data are presented as folds of mean values in saline rats, and error bars indicate SEM. Saline: *n* = 4–5; Meth: *n* = 4–5.

Finally, to examine whether the changes of mRNA expression observed in Experiment 3 were specific to neurons, we measured mRNA expression of *Grm3*, *Opcm1, and Usp9x* in DS homogenate and found no changes of all three genes on abstinence day 28 in Meth rats compared with saline rats (Figure 6C, *p* > 0.05).

## Discussion

We used the CTb-based FACS and examined mRNA expressions of candidate genes in striatonigral MSNs during incubation of Meth craving. First, we validated that in sorted CTb-positive neurons (striatonigral MSNs), there was a significant enrichment of *Drd1* mRNA expression and a significant depletion of *Drd2* mRNA expression compared with CTb-negative neurons (primarily non-striatonigral MSNs). Next, we found that on abstinence day 1, mRNA expression for *Gabrb3* decreased only in CTb-positive (but not CTb-negative) neurons of Meth rats compared with saline rats, while mRNA expression for *Usp7* decreased in all sorted DS neurons regardless of projection-specificity. On abstinence day 28, mRNA expression for *Grm3*, *Opcml*, and *Usp9x* in all sorted DS neurons (but not DS homogenate) increased in Meth rats compared with saline rats, regardless of projection-specificity. Together, these data demonstrated that incubation of Meth craving was associated with time-dependent, circuit-specific, and neuron-specific gene alterations involved in glutamatergic, GABAergic, opioidergic, and protein degradation signaling.

### The use of CTb-based FACS to profile projection-specific gene alterations

For the past two decades, FACS has emerged as a powerful approach to profile molecular alterations in distinct cell populations within the nervous systems (e.g., Lobo et al., 2006; Liu et al., 2014; Rubio et al., 2015; Li et al., 2015; Rubio et al., 2016; De Biase et al., 2017; Li et al., 2019; Zhang et al., 2021). These distinct cellular populations can be defined either by genetic tools (e.g., Lobo et al., 2006; Zhang et al., 2021) or antibodies that recognize specific cellular markers (e.g., Liu et al., 2014; Rubio et al., 2015; Li et al., 2015; Rubio et al., 2016; Li et al., 2019). We and others previously developed a FACS protocol to isolate behaviorally activated neurons using the fluorescence-conjugated antibodies against NeuN (the neuronal marker) and Fos (the neuronal activity marker) in fresh brain tissue (Liu et al., 2014; Rubio et al., 2015; Li et al., 2015). Subsequently, we adapted this FACS protocol to frozen brain tissue (Rubio et al., 2016; Li et al., 2019), which allows researchers to collect samples after complex behavioral procedures on the same experimental day before FACS.

Here, we modified this frozen-tissue FACS protocol to sort neurons anatomically defined by retrograde tracers like CTb-647. It is important to note that both NeuN and Fos, cellular markers used previously, are localized in the nucleus. Therefore, they can be used for subsequent FACS from frozen tissue because both are mostly retained in the nucleus after thawing. In our preliminary work (data not shown), we found that cytoplasmic makers (e.g., CTb-647) were easily lost during the thawing process, which left the cell population of interest unidentifiable during FACS. To ensure the retention of the CTb-647, we incubated the frozen striatal tissue with a low concentration of fixative (2% PFA) while the frozen tissue gradually thawed on ice in the following 40 min. We found that this modification successfully retained CTb-647 inside the cells, which was confirmed by the identification of a distinct CTb-positive population during FACS with significant enrichment and depletion of *Drd1* and *Drd2* mRNA expression, respectively. Taken together, our modified FACS protocol allows sorting cells from previously frozen brain tissue based on fluorescence-conjugated retrograde tracer CTb. This FACS protocol can also easily be applied to cells genetically labeled by various cytoplasmic localized fluorescent markers (e.g., green fluorescent protein) or labeled by antibodies against cytoplasmic cellular markers (e.g., parvalbumin).

However, there are two major limitations to using this CTb-based FACS protocol. First, CTb can label incorrect projections *via* passing fibers through the target region (Chen and Aston-Jones, 1995). On this front, we have attempted to isolate indirect-pathway neurons in DS after injecting CTb-647 into Gpe, only to find no enrichment of *Drd2* or depletion of *Drd1* mRNA expression in CTb-positive compared with CTb-negative neurons (data not shown). This data indicated the possibility of CTb labeling of direct-pathway neurons via their passing fiber through Gpe (Deng et al., 2006; Okamoto et al., 2020). To address this limitation, future studies can adapt this protocol to other retrograde tracing strategies (e.g., fluorescent latex microspheres) to minimize the entry of retrograde tracers into the passing fibers (Katz et al., 1984; Saleeba et al., 2019). The second limitation is the downstream application of RNA obtained from this FACS protocol. Similarly to our previous FACS protocols that involve cell fixation (Rubio et al., 2015; Li et al., 2015; Rubio et al., 2016; Li et al., 2019), the integrity of RNA obtained from our FACS protocol is low, with the RNA integrity number (RIN) ranging between 2 and 3. While we and others have successfully used PCR and microarray to measure gene expression of many candidate genes (Guez-Barber et al., 2011; Rubio et al., 2015; Li et al., 2015; Rubio et al., 2016; Li et al., 2019), the low integrity of RNA obtained here may limit the use of this protocol for unbiased next-generation sequencing studies (either bulk sequencing or single-cell sequencing), which usually requires the RIN number to be above 7. However, further studies can test if it is possible to perform sequencing studies combining this FACS protocol and the library preparation kits specifically designed for formalin-fixed paraffin-embedded tissue. Nonetheless, our FACS protocol is well-suited for profiling projection-specific gene alterations to answer hypothesis-driven questions.

### Circuit-dependent and neuron-specific gene alterations in DS during incubation of Meth craving

On abstinence day 1, we observed both circuit-dependent and circuit-independent decreases in mRNA expressions of *Gabrb3* and *Usp7*, respectively. These gene alterations are transient because we observed no statistical differences for these two genes in striatonigral neurons on abstinence day 28. On abstinence day 28, we observed circuit-independent but neuron-specific increases in mRNA expression of three candidate genes (*Grm3, Opcml, and Usp9x*) in DS. These gene alterations in DS were not observed on abstinence day 1, indicating that these gene alterations are time-dependent during incubation of Meth craving. Together, these data suggest that incubation of Meth craving is associated with transient perturbation of GABAergic transmission in striatonigral neurons during early abstinence, but time-dependent perturbation of glutamatergic transmission and opioidergic signaling in DS neurons after prolonged abstinence, and distinct perturbation of USP-associated protein degradation signaling in DS neurons throughout the abstinence.

Our data also identified neuron-specific *Grm3, Opcml, and Usp9x* as potential candidate gene targets for future studies on their roles in incubation of Meth craving. The finding on *Grm3* aligns with substantial evidence supporting the critical role of altered glutamatergic signaling in drug craving (Kalivas, 2009; Wolf, 2016; Caprioli et al., 2018). *Grm3* encodes metabotropic glutamate receptor 3 (mGlu3), which belongs to Group II metabotropic glutamate receptors. Unlike presynaptic-localized metabotropic glutamate receptor 2, mGlu3 is expressed on postsynaptic neurons and glia cells (Tamaru et al., 2001). In neurons, mGlu3 activates both the canonical G_i/*o*_-protein-mediated signaling and the non-canonical G_*q*_-protein-mediated signaling via metabotropic glutamate receptor 5 (mGlu5, Group I metabotropic glutamate receptor)(Di Menna et al., 2018; Dogra et al., 2021). Emerging evidence supports the role of mGlu3 across various psychiatric disorders, such as schizophrenia and addiction (Dogra et al., 2022). For example, mGlu3 knockout mice show increased Meth-induced CPP and sensitization (Busceti et al., 2021). In contrast, our data suggest that enhanced mGlu3 function in DS neurons may contribute to incubation of Meth craving, a hypothesis to test in future studies.

*Opcml* encodes opioid-binding protein/cell adhesion molecule-like, which belongs to the immunoglobulin-like family of cell adhesion molecules (Schofield et al., 1989). The genome-wide associate studies have linked the single nucleotide polymorphism of OPCML gene with schizophrenia (Athanasiu et al., 2010; Ji et al., 2010; Schol-Gelok et al., 2010; Zhang et al., 2019) and later studies using Opcml-deficient mice support this association (Zhang et al., 2019; Sun et al., 2024). However, the role of *Opcml* in addiction is completely unknown, and our finding for the first time linked *Opcml* to drug-associated behaviors. Similarly, the role of *Usp9x*, which encodes ubiquitin-specific peptidase 9x, in addiction is largely unknown. An early study found that the USP9x protein expression in prefrontal cortex increases after 21-day withdrawal from repeated non-contingent cocaine administration in rats (Xu et al., 2010), which is in line with our data here showing increased *Usp9x* mRNA expression in DS neurons after prolonged abstinence from Meth self-administration. In mice, USP9x is also identified as one of the top hub genes in nucleus accumbens after chronic non-contingent morphine administration (Lefevre et al., 2020). Moreover, decreased *Usp9x* mRNA expression was observed in the hippocampus of non-human primates with a history of chronic Meth or heroin expression (Choi et al., 2022). These findings together highlighted the potential role of USP9x across different brain regions in mediating drug-associated behaviors and, more broadly, the role of protein degradation signaling in the context of drug addiction (Massaly et al., 2013; Ren et al., 2013; Massaly et al., 2014; Werner et al., 2015; Werner et al., 2018; Werner et al., 2019).

One limitation of our study is that, due to our candidate gene approaches, we may have overlooked other gene targets in DS that are implicated in incubation of Meth craving. For example, a recent study showed changes in gene expressions in DS associated with neurotrophic factor or orexin signaling during incubation of Meth craving after punishment-imposed abstinence (Daiwile et al., 2024). It is also of note that the only genes we examined in CTb-negative neurons were those that showed differential expression in CTb-positive neurons because we aimed to test whether gene alterations observed in CTb-positive neurons generalized to all neurons regardless of circuit-specificity. Therefore, whether the mRNA expression of other candidate genes changed in CTb-negative neurons is unknown. However, since CTb-negative neurons contain mixed neuronal populations, significant gene alterations detected in CTb-negative neurons are not informative. A final limitation is that we did not examine gene alterations in DS homogenate on abstinence day 1, and therefore, whether gene alterations observed on abstinence day 1 are neuron-specific remains unknown.

## Conclusion

We developed a CTb-based FACS protocol, which allows for the isolation of projection-specific neurons from previously frozen brain tissue. Along with qPCR, our pipeline is well-suited for profiling projection-specific gene alterations using a candidate-gene approach after complex behavioral procedures. Using our pipeline to profile gene alterations in striatonigral neurons in DS, we found both circuit-dependent and circuit-independent gene alternation associated with glutamatergic, opioid signaling and protein-degradation signaling during incubation of Meth craving. Moreover, our data revealed time-dependent increases of novel neuron-specific candidate genes in DS neurons during abstinence, and future studies will examine the causal roles of these genes in DS neurons in Meth craving.

## Data Availability

The original contributions presented in the study are included in the article/Supplementary material, further inquiries can be directed to the corresponding author.

## References

[B1] Adhikary S. Caprioli D. Venniro M. Kallenberger P. Shaham Y. Bossert J. M. (2017). Incubation of extinction responding and cue-induced reinstatement, but not context- or drug priming-induced reinstatement, after withdrawal from methamphetamine. *Addict. Biol.* 22 977–990. 26989042 10.1111/adb.12386 PMC5023450

[B2] Athanasiu L. Mattingsdal M. Kähler A. K. Brown A. Gustafsson O. Agartz I. (2010). Gene variants associated with schizophrenia in a Norwegian genome-wide study are replicated in a large European cohort. *J. Psychiatr. Res.* 44 748–753. 10.1016/j.jpsychires.2010.02.002 20185149 PMC3224994

[B3] Bingol B. Sheng M. (2011). Deconstruction for reconstruction: the role of proteolysis in neural plasticity and disease. *Neuron* 69 22–32. 10.1016/j.neuron.2010.11.006 21220096

[B4] Bossert J. M. Wihbey K. A. Pickens C. L. Nair S. G. Shaham Y. (2009). Role of dopamine D(1)-family receptors in dorsolateral striatum in context-induced reinstatement of heroin seeking in rats. *Psychopharmacology (Berl)* 206 51–60. 10.1007/s00213-009-1580-x 19506837 PMC3145155

[B5] Busceti C. L. Ginerete R. P. Di Menna L. D’Errico G. Cisani F. Di Pietro P. (2021). Behavioural and biochemical responses to methamphetamine are differentially regulated by mGlu2 and mGlu3 metabotropic glutamate receptors in male mice. *Neuropharmacology* 196:108692. 10.1016/j.neuropharm.2021.108692 34217776

[B6] Caprioli D. Justinova Z. Venniro M. Shaham Y. (2018). Effect of novel allosteric modulators of metabotropic glutamate receptors on drug self-administration and relapse: a review of preclinical studies and their clinical implications. *Biol. Psychiatry* 84 180–192. 29102027 10.1016/j.biopsych.2017.08.018 PMC5837933

[B7] Caprioli D. Venniro M. Zhang M. Bossert J. M. Warren B. L. Hope B. T. (2017). Role of dorsomedial striatum neuronal ensembles in incubation of methamphetamine craving after voluntary abstinence. *J. Neurosci.* 37 1014–1027. 10.1523/JNEUROSCI.3091-16.2016 28123032 PMC5296775

[B8] Chen S. Aston-Jones G. (1995). Evidence that cholera toxin B subunit (CTb) can be avidly taken up and transported by fibers of passage. *Brain Res.* 674 107–111. 10.1016/0006-8993(95)00020-q 7773677

[B9] Cheron J. Beccari L. Hague P. Icick R. Despontin C. Carusone T. (2023). USP7/Maged1-mediated H2A monoubiquitination in the paraventricular thalamus: an epigenetic mechanism involved in cocaine use disorder. *Nat. Commun.* 14:8481. 10.1038/s41467-023-44120-2 38123574 PMC10733359

[B10] Choi M. R. Jin Y. B. Kim H. N. Lee H. Chai Y. G. Lee S. R. (2022). Differential gene expression in the hippocampi of nonhuman primates chronically exposed to methamphetamine, cocaine, or heroin. *Psychiatry Investig.* 19 538–550. 10.30773/pi.2022.0004 PMC9334808 35903056

[B11] Ciechanover A. (2005). Proteolysis: from the lysosome to ubiquitin and the proteasome. *Nat. Rev. Mol. Cell Biol.* 6 79–87. 15688069 10.1038/nrm1552

[B12] Corbit L. H. Nie H. Janak P. H. (2012). Habitual alcohol seeking: time course and the contribution of subregions of the dorsal striatum. *Biol. Psychiatry* 72 389–395. 10.1016/j.biopsych.2012.02.024 22440617 PMC3674580

[B13] Daiwile A. P. McCoy M. T. Ladenheim B. Subramaniam J. Cadet J. L. (2024). Incubation of methamphetamine craving in punishment-resistant individuals is associated with activation of specific gene networks in the rat dorsal striatum. *Mol. Psychiatry* 29 1990–2000. 10.1038/s41380-024-02455-2 38351172 PMC11408252

[B14] De Biase L. M. Schuebel K. E. Fusfeld Z. H. Jair K. Hawes I. A. Cimbro R. (2017). Local cues establish and maintain region-specific phenotypes of basal ganglia microglia. *Neuron* 95 341–356.e346. 10.1016/j.neuron.2017.06.020 28689984 PMC5754189

[B15] Deng Y. P. Lei W. L. Reiner A. (2006). Differential perikaryal localization in rats of D1 and D2 dopamine receptors on striatal projection neuron types identified by retrograde labeling. *J. Chem. Neuroanat.* 32 101–116. 10.1016/j.jchemneu.2006.07.001 16914290

[B16] Di Menna L. Joffe M. E. Iacovelli L. Orlando R. Lindsley C. W. Mairesse J. (2018). Functional partnership between mGlu3 and mGlu5 metabotropic glutamate receptors in the central nervous system. *Neuropharmacology* 128 301–313. 10.1016/j.neuropharm.2017.10.026 29079293 PMC6263139

[B17] Dogra S. Putnam J. Conn P. J. (2022). Metabotropic glutamate receptor 3 as a potential therapeutic target for psychiatric and neurological disorders. *Pharmacol. Biochem. Behav.* 221:173493. 10.1016/j.pbb.2022.173493 PMC9729465 36402243

[B18] Dogra S. Stansley B. J. Xiang Z. Qian W. Gogliotti R. G. Nicoletti F. (2021). Activating mGlu(3) metabotropic glutamate receptors rescues schizophrenia-like cognitive deficits through metaplastic adaptations within the hippocampus. *Biol. Psychiatry* 90 385–398. 10.1016/j.biopsych.2021.02.970 33965197 PMC8403106

[B19] Elkashef A. Vocci F. Hanson G. White J. Wickes W. Tiihonen J. (2008). Pharmacotherapy of methamphetamine addiction: an update. *Subst. Abus.* 29 31–49. 19042205 10.1080/08897070802218554 PMC2597382

[B20] Guez-Barber D. Fanous S. Golden S. A. Schrama R. Koya E. Stern A. L. (2011). FACS identifies unique cocaine-induced gene regulation in selectively activated adult striatal neurons. *J. Neurosci.* 31 4251–4259. 10.1523/JNEUROSCI.6195-10.2011 21411666 PMC3073079

[B21] Ji T. Wu Y. Wang H. Wang J. Jiang Y. (2010). Diagnosis and fine mapping of a deletion in distal 11q in two Chinese patients with developmental delay. *J. Hum. Genet.* 55 486–489. 10.1038/jhg.2010.51 20520618

[B22] Jiao D. L. Liu Y. Long J. D. Du J. Ju Y. Y. Zan G. Y. (2016). Involvement of dorsal striatal alpha1-containing GABAA receptors in methamphetamine-associated rewarding memories. *Neuroscience* 320 230–238. 10.1016/j.neuroscience.2016.02.001 26868969

[B23] Kalivas P. W. (2009). The glutamate homeostasis hypothesis of addiction. *Nat. Rev. Neurosci.* 10 561–572. 19571793 10.1038/nrn2515

[B24] Karila L. Weinstein A. Aubin H. J. Benyamina A. Reynaud M. Batki S. L. (2010). Pharmacological approaches to methamphetamine dependence: a focused review. *Br. J. Clin. Pharmacol.* 69 578–592. 20565449 10.1111/j.1365-2125.2010.03639.x PMC2883750

[B25] Katz L. C. Burkhalter A. Dreyer W. J. (1984). Fluorescent latex microspheres as a retrograde neuronal marker for in vivo and in vitro studies of visual cortex. *Nature* 310 498–500. 10.1038/310498a0 6205278

[B26] Krasnova I. N. Chiflikyan M. Justinova Z. McCoy M. T. Ladenheim B. Jayanthi S. (2013). CREB phosphorylation regulates striatal transcriptional responses in the self-administration model of methamphetamine addiction in the rat. *Neurobiol. Dis.* 58 132–143. 10.1016/j.nbd.2013.05.009 23726845 PMC3748236

[B27] Lefevre E. M. Pisansky M. T. Toddes C. Baruffaldi F. Pravetoni M. Tian L. (2020). Interruption of continuous opioid exposure exacerbates drug-evoked adaptations in the mesolimbic dopamine system. *Neuropsychopharmacology* 45 1781–1792. 10.1038/s41386-020-0643-x 32079024 PMC7608117

[B28] Leys C. Ley C. Klein O. Bernard P. Licata L. (2013). Detecting outliners: do not use standard deviation around the mean, use absolute deviation around the median. *J. Exp. Psychol.* 49 764–766.

[B29] Li X. Davis I. R. Lofaro O. M. Zhang J. Cimbro R. Rubio F. J. (2019). Distinct gene alterations between Fos-expressing striatal and thalamic neurons after withdrawal from methamphetamine self-administration. *Brain Behav.* 9:e01378. 10.1002/brb3.1378 31364821 PMC6749486

[B30] Li X. DeJoseph M. R. Urban J. H. Bahi A. Dreyer J. L. Meredith G. E. (2013). Different roles of BDNF in nucleus accumbens core versus shell during the incubation of cue-induced cocaine craving and its long-term maintenance. *J. Neurosci.* 33 1130–1142. 10.1523/JNEUROSCI.3082-12.2013 23325250 PMC3711541

[B31] Li X. Rubio F. J. Zeric T. Bossert J. M. Kambhampati S. Cates H. M. (2015). Incubation of methamphetamine craving is associated with selective increases in expression of Bdnf and trkb, glutamate receptors, and epigenetic enzymes in cue-activated fos-expressing dorsal striatal neurons. *J. Neurosci.* 35 8232–8244. 10.1523/JNEUROSCI.1022-15.2015 26019338 PMC4444544

[B32] Li X. Witonsky K. R. Lofaro O. M. Surjono F. Zhang J. Bossert J. M. (2018). Role of anterior intralaminar nuclei of thalamus projections to dorsomedial striatum in incubation of methamphetamine craving. *J. Neurosci.* 38 2270–2282. 29371321 10.1523/JNEUROSCI.2873-17.2018 PMC5830515

[B33] Liu Q. R. Rubio F. J. Bossert J. M. Marchant N. J. Fanous S. Hou X. (2014). Detection of molecular alterations in methamphetamine-activated Fos-expressing neurons from a single rat dorsal striatum using fluorescence-activated cell sorting (FACS). *J. Neurochem.* 128 173–185. 10.1111/jnc.12381 23895375 PMC3867527

[B34] Lobo M. K. (2009). Molecular profiling of striatonigral and striatopallidal medium spiny neurons past, present, and future. *Int. Rev. Neurobiol.* 89 1–35. 10.1016/S0074-7742(09)89001-6 19900613

[B35] Lobo M. K. Karsten S. L. Gray M. Geschwind D. H. Yang X. W. (2006). FACS-array profiling of striatal projection neuron subtypes in juvenile and adult mouse brains. *Nat. Neurosci.* 9 443–452. 10.1038/nn1654 16491081

[B36] Lu L. Grimm J. W. Hope B. T. Shaham Y. (2004). Incubation of cocaine craving after withdrawal: a review of preclinical data. *Neuropharmacology* 47 (Suppl. 1), 214–226. 15464139 10.1016/j.neuropharm.2004.06.027

[B37] Mabb A. M. Ehlers M. D. (2010). Ubiquitination in postsynaptic function and plasticity. *Annu. Rev. Cell Dev. Biol.* 26 179–210. 20604708 10.1146/annurev-cellbio-100109-104129 PMC3163670

[B38] Mandelbaum G. Taranda J. Haynes T. M. Hochbaum D. R. Huang K. W. Hyun M. (2019). Distinct cortical-thalamic-striatal circuits through the parafascicular nucleus. *Neuron* 102 636–652.e637. 10.1016/j.neuron.2019.02.035 30905392 PMC7164542

[B39] Martin D. Xu J. Porretta C. Nichols C. D. (2017). Neurocytometry: flow cytometric sorting of specific neuronal populations from human and rodent brain. *ACS Chem. Neurosci.* 8 356–367. 10.1021/acschemneuro.6b00374 28135061 PMC6385603

[B40] Massaly N. Dahan L. Baudonnat M. Hovnanian C. Rekik K. Solinas M. (2013). Involvement of protein degradation by the ubiquitin proteasome system in opiate addictive behaviors. *Neuropsychopharmacology* 38 596–604. 10.1038/npp.2012.217 23169349 PMC3572456

[B41] Massaly N. Frances B. Mouledous L. (2014). Roles of the ubiquitin proteasome system in the effects of drugs of abuse. *Front. Mol. Neurosci.* 7:99. 10.3389/fnmol.2014.00099 25610367 PMC4285073

[B42] Megan M. Chun Hui J. P. Alyna T. Alexandre A. G. Jee Hyun K. (2023). Past and current drug repurposing clinical trials to treat cognition in methamphetamine use: a scoping review of pharmacotherapy candidates. *Addict. Neurosci.* 5:100064.

[B43] Murray C. H. Loweth J. A. Milovanovic M. Stefanik M. T. Caccamise A. J. Dolubizno H. (2019). AMPA receptor and metabotropic glutamate receptor 1 adaptations in the nucleus accumbens core during incubation of methamphetamine craving. *Neuropsychopharmacology* 44 1534–1541. 10.1038/s41386-019-0425-5 31146278 PMC6785134

[B44] Murray J. E. Belin D. Everitt B. J. (2012). Double dissociation of the dorsomedial and dorsolateral striatal control over the acquisition and performance of cocaine seeking. *Neuropsychopharmacology* 37 2456–2466. 10.1038/npp.2012.104 22739470 PMC3442340

[B45] Okamoto S. Sohn J. Tanaka T. Takahashi M. Ishida Y. Yamauchi K. (2020). Overlapping projections of neighboring direct and indirect pathway neostriatal neurons to globus pallidus external segment. *iScience* 23:101409. 10.1016/j.isci.2020.101409 32877648 PMC7520896

[B46] Patrick G. N. (2006). Synapse formation and plasticity: recent insights from the perspective of the ubiquitin proteasome system. *Curr. Opin. Neurobiol.* 16 90–94. 10.1016/j.conb.2006.01.007 16427269

[B47] Paxinos G. Watson C. (2005). *The Rat Brain in Stereotaxic Coordinates*, 5 Edn. Amsterdam: Elsevier Academic Press.

[B48] Pena-Bravo J. I. Penrod R. Reichel C. M. Lavin A. (2019). Methamphetamine self-administration elicits sex-related changes in postsynaptic glutamate transmission in the prefrontal cortex. *eNeuro* 6 ENEURO.0401-18.2018. 10.1523/ENEURO.0401-18.2018 30693312 PMC6348447

[B49] Ren Z. Y. Liu M. M. Xue Y. X. Ding Z. B. Xue L. F. Zhai S. D. (2013). A critical role for protein degradation in the nucleus accumbens core in cocaine reward memory. *Neuropsychopharmacology* 38 778–790. 10.1038/npp.2012.243 23303053 PMC3672001

[B50] Rubio F. J. Li X. Liu Q. R. Cimbro R. Hope B. T. (2016). Fluorescence Activated Cell Sorting (FACS) and gene expression analysis of fos-expressing neurons from fresh and frozen rat brain tissue. *J. Vis. Exp.* 54358. 10.3791/54358 27685012 PMC5091963

[B51] Rubio F. J. Liu Q. R. Li X. Cruz F. C. Leao R. M. Warren B. L. (2015). Context-induced reinstatement of methamphetamine seeking is associated with unique molecular alterations in Fos-expressing dorsolateral striatum neurons. *J. Neurosci* 35 5625–5639. 10.1523/JNEUROSCI.4997-14.2015 25855177 PMC4388923

[B52] Saleeba C. Dempsey B. Le S. Goodchild A. McMullan S. (2019). A student’s guide to neural circuit tracing. *Front. Neurosci.* 13:897. 10.3389/fnins.2019.00897 31507369 PMC6718611

[B53] Scheyer A. F. Loweth J. A. Christian D. T. Uejima J. Rabei R. Le T. (2016). AMPA receptor plasticity in accumbens core contributes to incubation of methamphetamine craving. *Biol. Psychiatry* 80 661–670. 10.1016/j.biopsych.2016.04.003 27264310 PMC5050076

[B54] Schofield P. R. McFarland K. C. Hayflick J. S. Wilcox J. N. Cho T. M. Roy S. (1989). Molecular characterization of a new immunoglobulin superfamily protein with potential roles in opioid binding and cell contact. *EMBO J.* 8 489–495. 10.1002/j.1460-2075.1989.tb03402.x 2721489 PMC400831

[B55] Schol-Gelok S. Janssens A. C. Tiemeier H. Liu F. Lopez-Leon S. Zorkoltseva I. V. (2010). A genome-wide screen for depression in two independent Dutch populations. *Biol. Psychiatry* 68 187–196. 10.1016/j.biopsych.2010.01.033 20452571

[B56] Schwendt M. Reichel C. M. See R. E. (2012). Extinction-dependent alterations in corticostriatal mGluR2/3 and mGluR7 receptors following chronic methamphetamine self-administration in rats. *PLoS One* 7:e34299. 10.1371/journal.pone.0034299 22479593 PMC3315516

[B57] Shepard J. D. Bossert J. M. Liu S. Y. Shaham Y. (2004). The anxiogenic drug yohimbine reinstates methamphetamine seeking in a rat model of drug relapse. *Biol. Psychiatry* 55 1082–1089. 10.1016/j.biopsych.2004.02.032 15158427

[B58] Sun X. Meng H. Lu T. Yue W. Zhang D. Wang L. (2024). Mechanisms of glutamate receptors hypofunction dependent synaptic transmission impairment in the hippocampus of schizophrenia susceptibility gene Opcml-deficient mouse model. *Mol. Brain* 17:75. 10.1186/s13041-024-01148-9 39420375 PMC11488275

[B59] Tamaru Y. Nomura S. Mizuno N. Shigemoto R. (2001). Distribution of metabotropic glutamate receptor mGluR3 in the mouse CNS: differential location relative to pre- and postsynaptic sites. *Neuroscience* 106 481–503. 10.1016/s0306-4522(01)00305-0 11591452

[B60] Tien L. T. Ho I. K. (2011). Involvement of micro-opioid receptor in methamphetamine-induced behavioral sensitization. *Curr. Neuropharmacol.* 9 215–218. 10.2174/157015911795016949 21886593 PMC3137186

[B61] Wang G. Shi J. Chen N. Xu L. Li J. Li P. (2013). Effects of length of abstinence on decision-making and craving in methamphetamine abusers. *PLoS One* 8:e68791. 10.1371/journal.pone.0068791 23894345 PMC3722210

[B62] Wang J. Lanfranco M. F. Gibb S. L. Yowell Q. V. Carnicella S. Ron D. (2010). Long-lasting adaptations of the NR2B-containing NMDA receptors in the dorsomedial striatum play a crucial role in alcohol consumption and relapse. *J. Neurosci.* 30 10187–10198. 10.1523/JNEUROSCI.2268-10.2010 20668202 PMC2950094

[B63] Werner C. T. Milovanovic M. Christian D. T. Loweth J. A. Wolf M. E. (2015). Response of the ubiquitin-proteasome system to memory retrieval after extended-access cocaine or saline self-administration. *Neuropsychopharmacology* 40 3006–3014. 26044907 10.1038/npp.2015.156 PMC4864635

[B64] Werner C. T. Mitra S. Martin J. A. Stewart A. F. Lepack A. E. Ramakrishnan A. (2019). Ubiquitin-proteasomal regulation of chromatin remodeler INO80 in the nucleus accumbens mediates persistent cocaine craving. *Sci. Adv.* 5:eaay0351. 10.1126/sciadv.aay0351 31633032 PMC6785264

[B65] Werner C. T. Viswanathan R. Martin J. A. Gobira P. H. Mitra S. Thomas S. A. (2018). E3 ubiquitin-protein ligase SMURF1 in the nucleus accumbens mediates cocaine seeking. *Biol. Psychiatry* 84 881–892. 10.1016/j.biopsych.2018.07.013 30158054 PMC6260585

[B66] Wolf M. E. (2016). Synaptic mechanisms underlying persistent cocaine craving. *Nat. Rev. Neurosci.* 17 351–365. 27150400 10.1038/nrn.2016.39 PMC5466704

[B67] Xie X. Zhuang D. Gu J. Wu T. Shen W. Li L. (2023). Association of GABA receptor delta subunit gene variations with increased risk of methamphetamine dependence. *Neurosci. Lett.* 800:137137. 10.1016/j.neulet.2023.137137 36804572

[B68] Xu Z. Xia B. Gong Q. Bailey J. Groves B. Radeke M. (2010). Identification of a deubiquitinating enzyme as a novel AGS3-interacting protein. *PLoS One* 5:e9725. 10.1371/journal.pone.0009725 20305814 PMC2840025

[B69] Zhang Z. Ye M. Li Q. You Y. Yu H. Ma Y. (2019). The schizophrenia susceptibility gene OPCML regulates spine maturation and cognitive behaviors through Eph-cofilin signaling. *Cell Rep.* 29 49–61.e47. 10.1016/j.celrep.2019.08.091 31577955

[B70] Zhang Z. Zhou J. Tan P. Pang Y. Rivkin A. C. Kirchgessner M. A. (2021). Epigenomic diversity of cortical projection neurons in the mouse brain. *Nature* 598 167–173. 34616065 10.1038/s41586-021-03223-w PMC8494636

