# Supplementary material for: Profiling gene alterations in striatonigral neurons associated with incubation of methamphetamine craving by cholera toxin subunit B-based fluorescence-activated cell sorting
Source: Front Cell Neurosci. 2025 Feb 12;19:1542508. doi: 10.3389/fncel.2025.1542508 (PMC11860961; doi:10.3389/fncel.2025.1542508)
Supplement: Supplementary file 1 [file Table_1.docx]

12-6-24

Research article

**SUPPLEMENTAL MATERIAL**

**Profiling gene alterations in striatonigral neurons associated with incubation of methamphetamine craving by retrograde tracer-based fluorescence-activated cell sorting**

^1^Rachel D. Altshuler, ^1^Megan A.M. Burke, ^1^Kristine T. Garcia, ^2^Kenneth Class, ^3#^Raffaello Cimbro and ^1,4*^Xuan Li

Affiliations:

^1^Department of Psychology, University of Maryland College Park, College Park, MD 20742

^2^Department of Cell Biology and Molecular Genetics, University of Maryland College Park, College Park, MD 20742

^3^ Division of Rheumatology, Johns Hopkins University School of Medicine, Baltimore, Maryland, USA

^4^Program in Neuroscience and Cognitive Science, University of Maryland College Park, College Park, MD 20742

^#^ Current address: Dynamic Omics, Centre of Genomic Research, Discovery Sciences, BioPharmaceuticals R&D, AstraZeneca, Gaithersburg, USA

**Table S1. Statistical analysis**

| **Figure** | **Test** | **F- Value** | ***p*-value** | **Partial Eta^2^** |
| --- | --- | --- | --- | --- |
| **Fig. 2B**  Exp. 2 Training | Infusion  Training day (within)  Group (between)  Training day * Group  Lever Presses  Lever (within)  Training day (within)  Group (between)  Training day * Lever  Lever * Group  Training day * Group  Training day * Lever * Group | F_9,135_= 1.192  F_1,15_= 50.779  F_9,135_= 6.934  F_1,15_= 141.644  F_9,135_= 8.845  F_1,15_= 2.911  F_9,135_= 0.321  F_1,15_= 58.398  F_9,135_= 8.994  F_9,135_= 1.392 | 0.305  <0.001*  <0.001*  <0.001*  <0.001*  0.109  0.967  <0.001*  <0.001*  0.198 | 0.074  0.772  0.316  0.904  0.371  0.163  0.021  0.796  0.375  0.085 |
| **Fig. 2E**  Exp. 2 Number and percentage of CTb+ neurons | Count  Saline vs. Meth  Percentage  Saline vs. Meth | t_15_= -0.716  t_15_= -0.423 | 0.485  0.678 |  |
| **Fig. 2F**  Exp. 2 Validation of cell types | Drd1  Cell type (within)  Drug (between)  Cell type * Drug  *Post hoc (within)*  Saline  Meth  Drd2  Cell type (within)  Drug (between)  Cell type * Drug  *Post hoc (within)*  Saline  Meth | F_1,15_= 178.872  F_1,15_= 1.419  F_1,15_= 2.838  t_7_= 8.531  t_8_= 11.11  F_1,15_= 67.335  F_1,15_= 0.164  F_1,15_= 0.055  t_7_= 12.97  t_8_= 4.692 | <0.001*  0.252  0.113  <0.001*  <0.001*  <0.001*  0.691  0.818  <0.001*  <0.001* | 0.923  0.086  0.159  0.818  0.011  0.004 |
| **Fig. 3A**  Exp. 2 Expression of glutamate receptor genes in CTb+ neurons | Drug (between)  *Gria1*  *Gria2*  *Gria3*  *Gria4*  *Grin1*  *Grin2a*  *Grin2b*  *Grm1*  *Grm2*  *Grm3*  *Grm4*  *Grm5* | t_13_= 0.319  t_14_= 0.240  t_13_= 0.896  t_15_= 0.841  t_15_= 0.180  t_15_= 1.005  t_14_= 1.751  t_14_= 1.683  t_13_= 2.176  t_15_= 1.097  t_14_= 0.043  t_15_= 0.609 | 0.755  0.814  0.387  0.414  0.859  0.331  0.102  0.114  0.049  0.290  0.966  0.552 |  |
| **Fig. 3B**  Exp. 2 Expression of GABA and opioid receptor genes in CTb+ neurons | Drug (between)  *Gabra1*  *Gabra3*  *Gabra5*  *Gabrb2*  *Gabrb3*  *Gabrg2*  *Gabbr2*  *Oprd1*  *Oprk1*  *Oprm1*  *Opcml* | t_15_= 1.886  t_14_= 0.631  t_14_= 0.698  t_15_= 1.681  t_14_= 4.275  t_14_= 1.420  t_15_= 0.657  t_14_= 1.182  t_14_= 0.595  t_15_= 2.592  t_15_= 1.737 | 0.079  0.538  0.497  0.113  <0.001*  0.178  0.521  0.257  0.561  0.020  0.103 |  |
| **Fig. 3C**  Exp. 2 Expression of USP genes in CTb+ neurons | Drug (between)  *Usp5*  *Usp7*  *Usp9x*  *Usp11*  *Usp14*  *Usp15*  *Usp22*  *Usp34*  *Usp47*  *Usp48* | t_14_= 0.921  t_14_= 3.335  t_14_= 1.754  t_14_= 0.529  t_14_= 1.333  t_14_= 1.548  t_15_= 1.923  t_15_= 1.716  t_13_= 2.351  t_15_= 0.084 | 0.372  0.005*  0.101  0.605  0.204  0.144  0.074  0.107  0.035  0.935 |  |
| **Fig. 3E**  Exp. 2 Expression of select genes in CTb- neurons | Drug (between)  *Gabrb3*  *Usp7* | t_14_= 1.606  t_15_= 2.200 | 0.131  0.044* |  |
| **Fig. 4B**  Exp. 3 Training | Infusion  Training day (within)  Group (between)  Training day * Group  Lever Presses  Lever (within)  Training day (within)  Group (between)  Training day * Lever  Lever * Group  Training day * Group  Training day * Lever * Group | F_9,144_= 3.342  F_1,16_= 52.407  F_9,144_= 15.262  F_1,16_= 164.089  F_9,144_= 1.290  F_1,16_= 20.134  F_9,144_= 3.300  F_1,16_= 78.498  F_9,144_= 7.040  F_9,144_= 7.422 | <0.001*  <0.001*  <0.001*  <0.001*  0.247  <0.001*  0.001*  <0.001*  <0.001*  <0.001* | 0.173  0.766  0.488  0.911  0.075  0.557  0.171  0.831  0.306  0.317 |
| **Fig. 4D**  Exp. 3 Number and percentage of CTb+ neurons | Count  Saline vs. Meth  Percentage  Saline vs. Meth | t_16_= 0.834  t_16_= 0.558 | 0.416  0.584 |  |
| **Fig. 4E**  Exp. 3 Validation of cell types | *Drd1*  Cell type (within)  Drug (between)  Cell type * Drug  Post hoc (Within)  Saline  Meth  *Drd2*  Cell type (within)  Drug (between)  Cell type * Drug  Post hoc (within)  Saline  Meth | F_1,16_= 108.206  F_1,16_= 0.048  F_1,16_= 0.221  t_8_= 9.673  t_8_= 6.004  F_1,16_= 99.526  F_1,16_= 1.919  F_1,16_= 1.887  t_8_= 5.590  t_8_= 8.885 | <0.001*  0.829  0.645  <0.001*  <0.001*  <0.001*  0.185  0.188  <0.001*  <0.001* | 0.871  0.003  0.014  0.862  0.107  0.106 |
| **Fig. 5A**  Exp. 3 Expression of glutamate receptor genes in CTb+ neurons | Drug (between)  *Gria1*  *Gria2*  *Gria3*  *Gria4*  *Grin1*  *Grin2a*  *Grin2b*  *Grm1*  *Grm2*  *Grm3*  *Grm4*  *Grm5* | t_16_= 0.357  t_15_= 0.015  t_16_= 1.365  t_14_= 0.520  t_16_= 0.421  t_16_= 0.209  t_15_= 0.193  t_16_= 0.203  t_11_= 2.722  t_14_= 5.358  t_16_= 1.502  t_16_= 2.223 | 0.726  0.988  0.191  0.612  0.680  0.837  0.849  0.841  0.020  <0.001*  0.153  0.041 |  |
| **Fig. 5B**  Exp. 3 Expression of GABA and opioid receptor genes in CTb+ neurons | Drug (between)  *Gabra1*  *Gabra3*  *Gabra5*  *Gabrb2*  *Gabrb3*  *Gabrg2*  *Gabbr2*  *Oprd1*  *Oprk1*  *Oprm1*  *Opcml* | t_14_= 0.635  t_12_= 1.212  t_15_= 0.165  t_14_= 1.552  t_16_= 0.781  t_15_= 0.302  t_16_= 0.059  t_16_= 0.285  t_15_= 0.487  t_16_= 0.347  t_14_= 3.602 | 0.536  0.249  0.871  0.143  0.446  0.767  0.953  0.780  0.633  0.733  0.003* |  |
| **Fig. 5C**  Exp. 3 Expression of USP genes in CTb+ neurons | Drug (between)  *Usp5*  *Usp7*  *Usp9x*  *Usp11*  *Usp14*  *Usp15*  *Usp22*  *Usp34*  *Usp47*  *Usp48* | t_16_= 0.020  t_15_= 2.297  t_15_= 2.995  t_14_= 0.776  t_14_= 0.770  t_15_= 0.592  t_15_= 0.353  t_16_= 1.502  t_16_= 0.838  t_15_= 1.560 | 0.985  0.036  0.009*  0.451  0.454  0.563  0.729  0.153  0.415  0.140 |  |
| **Fig. 5E**  Exp. 3 Expression of select genes in CTb- neurons | Drug (between)  *Grm3*  *Opcml*  *Usp9x* | t_15_= 2.827  t_15_= 2.453  t_15_= 2.832 | 0.013*  0.027*  0.013* |  |
| **Fig. 6B**  Exp. 4 Training | Infusion  Training day (within)  Group (between)  Training day * Group  Lever Presses  Lever (within)  Training day (within)  Group (between)  Training day * Lever  Lever * Group  Training day * Group  Training day * Lever * Group | F_9,72_= 2.776  F_1,8_= 27.186  F_9,72_= 4.762  F_1,8_= 46.620  F_9,72_= 3.124  F_1,8_= 15.034  F_9,72_= 3.475  F_1,8_= 28.984  F_9,72_= 4.562  F_9,72_= 3.030 | 0.008*  <0.001*  <0.001*  <0.001*  0.003*  0.005*  0.001*  <0.001*  <0.001*  0.004* | 0.258  0.773  0.373  0.854  0.281  0.653  0.303  0.784  0.363  0.275 |
| **Fig. 6C**  Exp. 4 Expression of select genes in DS homogenate | Drug (between)  *Grm3*  *Opcml*  *Usp9x* | t_8_= 0.571  t_7_= 0.832  t_8_= 0.280 | 0.584  0.433  0.787 |  |
